# Early CMV DNAemia during letermovir prophylaxis predicts lower risk of late CMV infection and is associated with enhanced T-cell immunity after alloHSCT

**DOI:** 10.1038/s41598-025-27490-z

**Published:** 2025-11-20

**Authors:** Timothé Duplessix, Ben-Niklas Baermann, Alexander Sebastian Hölscher, Inga Tometten, Felicitas Schulz, Stefanie Munder, Duyen Bao Le, Paul Jäger, Kathrin Nachtkamp, Sascha Dietrich, Jörg Timm, Guido Kobbe, Nadine Lübke

**Affiliations:** 1 Institute of Virology, University Hospital Düsseldorf, Medical Faculty, Heinrich-Heine-University, Düsseldorf, Germany; 2 Department of Hematology, Oncology and Clinical Immunology, Center of Integrated Oncology ABCD, University Hospital Düsseldorf, Heinrich-Heine-University, Düsseldorf, Germany; 3 Institute for Transplantation Diagnostics and Cellular Therapy, University Hospital Düsseldorf, Heinrich-Heine-University, Düsseldorf, Germany

**Keywords:** CMV infection, letermovir prophylaxis, T-cell immunity, ELISpot analysis, alloHSCT, Diseases, Immunology, Medical research, Microbiology

## Abstract

CMV-specific immune monitoring may support individualized risk stratification after allogeneic hematopoietic stem cell transplantation (alloHSCT), but its role in the era of letermovir prophylaxis remains unclear. In this prospective single-center study, 45 CMV-seropositive alloHSCT recipients receiving letermovir prophylaxis for 100 days post-transplant were followed for 12 months. CMV-specific T-cell responses to IE-1 and pp65 were assessed by IFN-γ ELISpot on days 100 and 200, and at the time of CMV infection and follow-up. Early CMV infection occurred in 18/45 patients (40%) during prophylaxis, of which 16 (88.9%) were transient “blips” and required no pre-emptive therapy. In univariate logistic regression, early CMV infection was associated a significantly lower risk of late CMV infection (*p* = 0.0044). IE-1-specific responses at day 100 were significantly higher in patients without late infection (median 27.5 vs. 5.0 SFC/250,000 PBMCs; *p* = 0.0056), and in those with early CMV infection (*p* = 0.0351). T-cell responses were already detectable at the time of CMV infection and remained stable thereafter. In conclusion, early abortive CMV DNAemia during letermovir prophylaxis appears to induces rapid and durable T-cell immunity. Immune monitoring may not be required in these patients, whereas ELISpot testing at day 100 may guide risk-adapted management in those without early CMV exposure.

**Supplementary Information:**

The online version contains supplementary material available at 10.1038/s41598-025-27490-z.

## Introduction

Human Cytomegalovirus (CMV) is still considered as one of the most dangerous viral complications in patients undergoing allogenic hematopoietic stem-cell transplantation (alloHSCT) and is associated with high morbidity and mortality^1^. The management of CMV infection following HSCT is still a challenge and traditionally includes two strategies: pre-emptive therapy (PET) with (val)ganciclovir or foscarnet, or an universal prophylaxis using letermovir^2^.

Letermovir, a terminase-complex inhibitor recently introduced for CMV IgG positive recipients, significantly reduces the number of clinically significant CMV infections (csCMVi) when administered during the first 100 days after alloHSCT^3,4^. It also demonstrates a lower incidence of toxicities (myelotoxicities or nephrotoxicity’s) compared to drugs used for preemptive Therapy PET)^3,5,6^. While letermovir is effective in reducing early-onset csCMVi, the emergence of late-onset csCMVi after discontinuation at day 100 has become a common concern, potentially leading to more late CMV-associated complications, particularly when the patients are monitored less frequently^3,7–9^.

As previous studies have shown, impaired CMV specific CD4^+^ and CD8^+^ T-cell reconstitution, along with GvHD and CMV donor status, are major risk factors for late csCMVi^9–12^. It is well established that letermovir delays the reconstitution of CMV-mediated T-cell immunity^2,13^, likely due to insufficient exposure of the immune system to CMV antigens, such as immediate early 1 (IE-1) and phosphoprotein 65 (pp65)^14^. However, as letermovir inhibits the terminase complex rather than the DNA-polymerase, noninfectious particles may be released from infected cells, potentially triggering T-cell responses and causing abortive infections, so called “blips”^14^.

Previous studies have suggested that monitoring CMV-specific T-cell immunity in seropositive patients after alloHSCT using immunological assays can help identify those at risk for csCMVi and offer favorable prognostic value for late CMVi^15–18^.

These assays measure the CD8^+^ and CD4^+^ immune response to CMV-specific antigens like IE-1 and pp65 by detecting interferon-gamma (IFN-γ) production, commonly using IFN-γ release assays (IGRA) such as T-SPOT.CMV (Oxford Immunotec, Revvity (ELISpot-based))^19^.

Against this background, the study had two main objectives: first, to evaluate whether CMV-specific T-cell responses, measured by IFN-γ ELISpot assays at the end of letermovir prophylaxis (day 100) and at day 200 after alloHSCT, are associated with the risk of late CMVi and late csCMVi; and second, to characterize the kinetics and durability of CMV-specific T-cell reconstitution over time following alloHSCT.

## Methods

### Ethical statement

The study was approved by the Ethics Committee of the Medical Faculty, Heinrich-Heine-University Düsseldorf (reference number: 2022-2147_1, 24.11.22), and all patients provided written informed consent prior to enrollment. All methods were performed in accordance with the relevant guidelines and regulations. In addition, the study was registered at the German Clinical Trials Register (DRKS-ID: DRKS00031287).

### Study population

Forty-five adult CMV-seropositive patients undergoing allogeneic hematopoietic stem cell transplantation (alloHSCT), including matched related, matched unrelated, and haploidentical donors at the University Hospital Düsseldorf between October 2022 and December 2023 were enrolled in this study (Table 1). All patients were monitored post-transplant for virological and immunological parameters, including CMV DNAemia, CMV-specific T-cell immunity, and absolute lymphocyte counts for an observation period of 12 months.

**Table 1:** Baseline characteristics of the study population according to CMV serostatus. Summary of baseline clinical, demographic, virological, and transplant-related characteristics of the study cohort (n = 45), stratified by donor/recipient CMV serostatus, including diagnosis, donor type, conditioning intensity, GvHD prophylaxis, and CMV serostatus. Percentages refer to the respective subgroup totals unless otherwise indicated. D, Donor; R, recipient; PBSC, peripher blood stem cells; MUD, matched-unrelated donor; MRD, matched- related donor; Haplo, haploidentical; RIC, reduced-intensity conditioning regimen; MAC, myeloablative conditioning regimen; GvHD, graft-versus-host-disease; MMF, mycophenolate-mophetil; CNI, calcineurin-inhibitors; PTCy, post-transplant cyclophosphamide.

		CMV status	
Characteristics	Total: N = 45 (%)	D + /R + : N = 27 (%)	D-/R + : N = 18 (%)
Sex			
Female	23 (51)	14 (52)	9 (50)
Male	22 (49)	13 (48)	9 (50)
Age			
Age (median in years, IQR)	59 (53–64.5)	58 (54–64.5)	61 (55.5–66.5)
Diagnosis			
Myeloid diseases	35 (78)	20 (74)	15 (83)
Lymphoid diseases	10 (22)	7 (26)	3 (17)
Graft source			
PBSC	44 (98)	26 (96)	18 (100)
Bone marrow	1 (2)	1 (4)	0 (0)
Donor type			
MUD	28 (62)	19 (70.4)	9 (50)
MRD	4 (8)	2 (7.4)	2 (11)
Haplo	13 (30)	6 (22.2)	7 (39)
Conditioning Regimen			
RIC	34 (76)	17 (63)	17 (94)
MAC	11 (24)	10 (37)	1 (6)
GvHD prophylaxis			
MMF + CNI only	4 (9)	2 (7.4)	2 (11.1)
MMF + CNI + ATG	28 (62)	19 (70.4)	9 (50)
MMF + CNI + PTCy	13 (29)	6 (22.2)	7 (38.9)
GvHD			
Acute GvHD (any grade)	25 (56%)	11 (41)	14 (77.8)
Chronic GvHd	4 (9%)	3 (11)	1 (5.6)

Inclusion criteria were age > 18 years, CMV-seropositivity at the time of alloHSCT, receipt of letermovir prophylaxis following transplantation, and provision of written informed consent. Clinical follow-up continued for 100 days after cessation of letermovir prophylaxis.

Demographic and transplant-related data, including CMV serostatus of donor and recipient, type of transplant, conditioning regimen, underlying malignancy, as well as the incidence and prophylaxis of acute (aGvHD) and chronic graft-versus-host disease (cGvHD) were collected from medical records. Due to incomplete documentation in some cases, grading of acute GvHD severity was not consistently available and therefore not included in the analysis. Information on CMV DNAemia and antiviral therapy administered in case of CMV infection was also recorded.

### Virological surveillance and classification of CMV infection

Patients were monitored for CMV DNA by quantitative PCR at least weekly until day 100 post-HSCT during letermovir prophylaxis, and thereafter every two weeks during follow-up visits or weekly in case of clinically significant CMV infection (csCMVi) or clinical suspicion, ensuring continuous virological surveillance throughout the study period.

Plasma DNA was extracted and analyzed using the cobas® CMV Test on the cobas® 5800 platform (Roche Molecular Systems). Viral loads were reported in IU/ml, with a lower limit of detection of 20.6 IU/ml. Pre-emptive antiviral therapy (valganciclovir and CMV-specific intravenous immunoglobulin, IVIgG) was initiated in cases of CMV-DNA plasma concentration ≥ 500 IU/ml in two consecutive samples. All CMV PCR analyses were performed in the same central laboratory at the University Hospital Düsseldorf with the same CMV quantification assay, ensuring methodological consistency and comparability of viral load measurements across all patients.

In line with the recent consensus definitions by Ljungman et al. (2024), CMV infections (CMVi) in transplant recipients were categorized into CMV DNAemia and clinically significant CMV infection (csCMVi)^20^. CMV infection (CMVi) was defined as any evidence of CMV replication, irrespective of clinical presentation. CMV DNAemia refers to the detection of CMV DNA in plasma by quantitative PCR, independent of clinical symptoms or treatment indication. Clinically significant CMV infection (csCMVi) was defined as CMV DNAemia requiring pre-emptive antiviral therapy or associated with CMV-related symptoms.

To indicate timing relative to letermovir prophylaxis, CMV infection was further specified as early CMVi, occurring during letermovir prophylaxis, and late CMVi, occurring after discontinuation of prophylaxis.

### Longitudinal monitoring of CMV-specific T-cell immunity

**Fig. 1:**
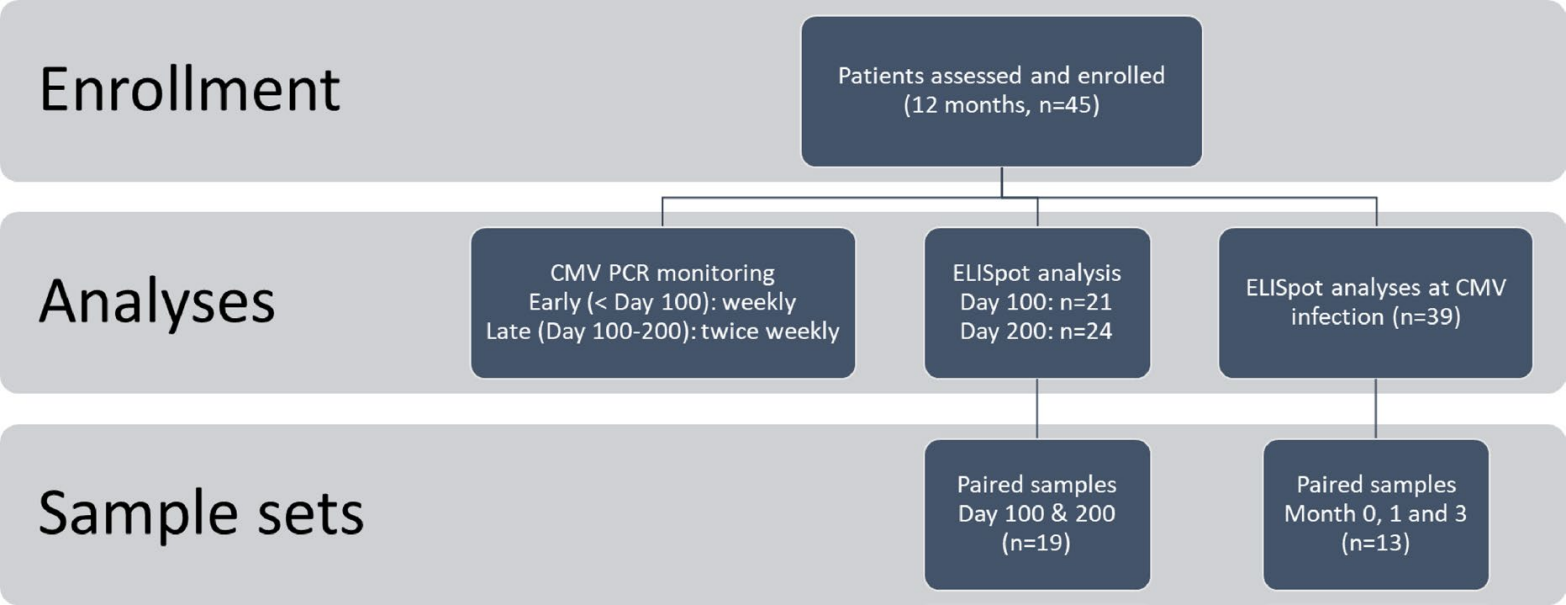
Overview of study enrollment, analyses, and sample sets. Flow diagram illustrating the study design and available analyses. A total of 45 patients were assessed and enrolled over a 12-month period. Virological monitoring was performed by CMV PCR weekly during the early phase (< day 100 post-HSCT) and twice weekly during the late phase (day 100–200). ELISpot analyses were conducted at day 100 (n = 21) and day 200 (n = 24), as well as after CMV DNAemia (n = 39). Two longitudinal sample sets were evaluated: paired samples collected at day 100 and 200 (n = 19), and paired samples following CMV DNAemia obtained at month 0, 1, and 3 (n = 14). MV, cytomegalovirus; DNAemia, detection of CMV DNA in plasma; HSCT, hematopoietic stem-cell transplantation; ELISpot, enzyme-linked immunospot assay.

CMV-specific T-cell immunity was monitored by IFN-γ ELISpot assay at predefined time points during the 12-month observation period: whenever possible on days 100 and 200 post-HSCT, at the time of CMV infection (CMVi), and at 1 and 3 months thereafter (± 5 working days). An overview of study design, CMV PCR monitoring, and availability of immunological samples is provided in Fig. 1.

ELISpot analyses were performed in a predefined translational subset of patients, depending on PBMC availability, assay capacity, and clinical eligibility. Patients with active acute GvHD or receiving systemic corticosteroids ≥ 1 mg/kg prednisolone equivalent at day 100 were excluded from immunological analyses to avoid confounding immune suppression. Consequently, ELISpot data were available for 21 patients at day 100 and 24 patients at day 200, including 19 patients with paired samples. In addition, 39 patients were analyzed at the time of CMV infection, and 13 patients contributed longitudinal samples (month 0, 1, and 3).

Peripheral blood mononuclear cells (PBMCs) were isolated from heparinized blood samples using the T-cell Select Kit (Revvity) and the Autopure-20B device (Allsheng), based on positive magnetic selection according to the manufacturer’s instructions^21^. All samples were processed within 24 h and analyzed using the CMV-specific ELISpot assay (Oxford Immunotec / Revvity), following validated standard procedures^22^. Each assay was performed in duplicate per patient and time point. T-cell responses were quantified as spot-forming cells (SFC) per 250,000 PBMCs, using an automated Bioreader ELISpot reader (BIOREADER® 7000-E, BIOSYS). Background activity was subtracted using medium-only controls; phytohemagglutinin (PHA) served as positive control.

### Statistical analyses

Descriptive statistics were used to summarize demographic and clinical characteristics, including counts, percentages, means, and standard deviations. All non-regression-based analyses were performed using GraphPad Prism (version 10.4.0).

Comparisons of ELISpot results between groups were conducted using the non-parametric Mann–Whitney U test. Receiver operating characteristic (ROC) curve analyses were performed to assess the predictive performance of IE-1 and pp65-specific T-cell responses for late CMV infection. Optimal cut-off values were determined using the Youden index, and area under the curve (AUC) values were reported with 95% confidence intervals.

Associations between dichotomized ELISpot results and CMV outcomes were evaluated using Fisher’s exact test.

Univariate logistic regression analyses were performed to explore associations between clinical and transplant-related variables and the occurrence of late CMV infection. Odds ratios (OR) with 95% confidence intervals (CI) and* p*-values were calculated for each variable separately. Given the limited number of late CMV infection events, no formal multivariable model was applied to avoid overfitting.

For variables with more than two categories, dummy coding was applied, using haploidentical donor and MMF + CNI as the respective reference groups, except for donor type, where MUD (matched unrelated donor) was used as the reference because this category represented the largest and most homogeneous subgroup within the cohort.

Cumulative incidence of late CMV infection was estimated using Kaplan–Meier analysis, with comparisons between groups performed using the log-rank test.

Regression analyses were conducted using R (version 4.3.2) and SPSS (version 29). All statistical tests were two-sided, and *p*-values < 0.05 were considered statistically significant.

## Results

A total of 45 patients undergoing allogeneic HSCT were enrolled and followed for 12 months (Table 1). Baseline characteristics are shown for the total study population as well as separately for CMV-seropositive recipients with seropositive (D + /R + , n = 27) and seronegative (D − /R + , n = 18) donors. The two subgroups were generally comparable regarding age, sex, and underlying disease. However, D + /R + patients more frequently received myeloablative conditioning (37% vs. 6%) and transplants from matched unrelated donors (70% vs. 50%), while D-/R + recipients more often underwent haploidentical transplantation (39% vs. 22%) and tended to experience acute GvHD more frequently (78% vs. 41%). The use of reduced-intensity conditioning was more common in D-/R + recipients (94% vs. 63%). GvHD prophylaxis strategies were distributed accordingly. All patients received letermovir prophylaxis starting within the first 27 days post-transplant.

In six patients (13%), low-level CMV DNAemia was detected prior to the initiation of letermovir prophylaxis. These episodes were transient and did not require pre-emptive antiviral therapy, and letermovir was started shortly thereafter. The standard dose of 480 mg was administered to 41 patients receiving tacrolimus for GvHD prophylaxis. Four patients received a reduced dose of 240 mg due to concomitant ciclosporin A treatment. The median duration of letermovir prophylaxis was 104 days (IQR 99–136, range 89–150 days). In seven patients (15.5%), prophylaxis was extended beyond day 100 and discontinued between day 145 and day 150 post-HSCT, primarily due to recurrent CMV DNAemia. Two patients died from non-CMV-related causes, one before day 100 and one between days 100 and 200.

**Fig. 2:**
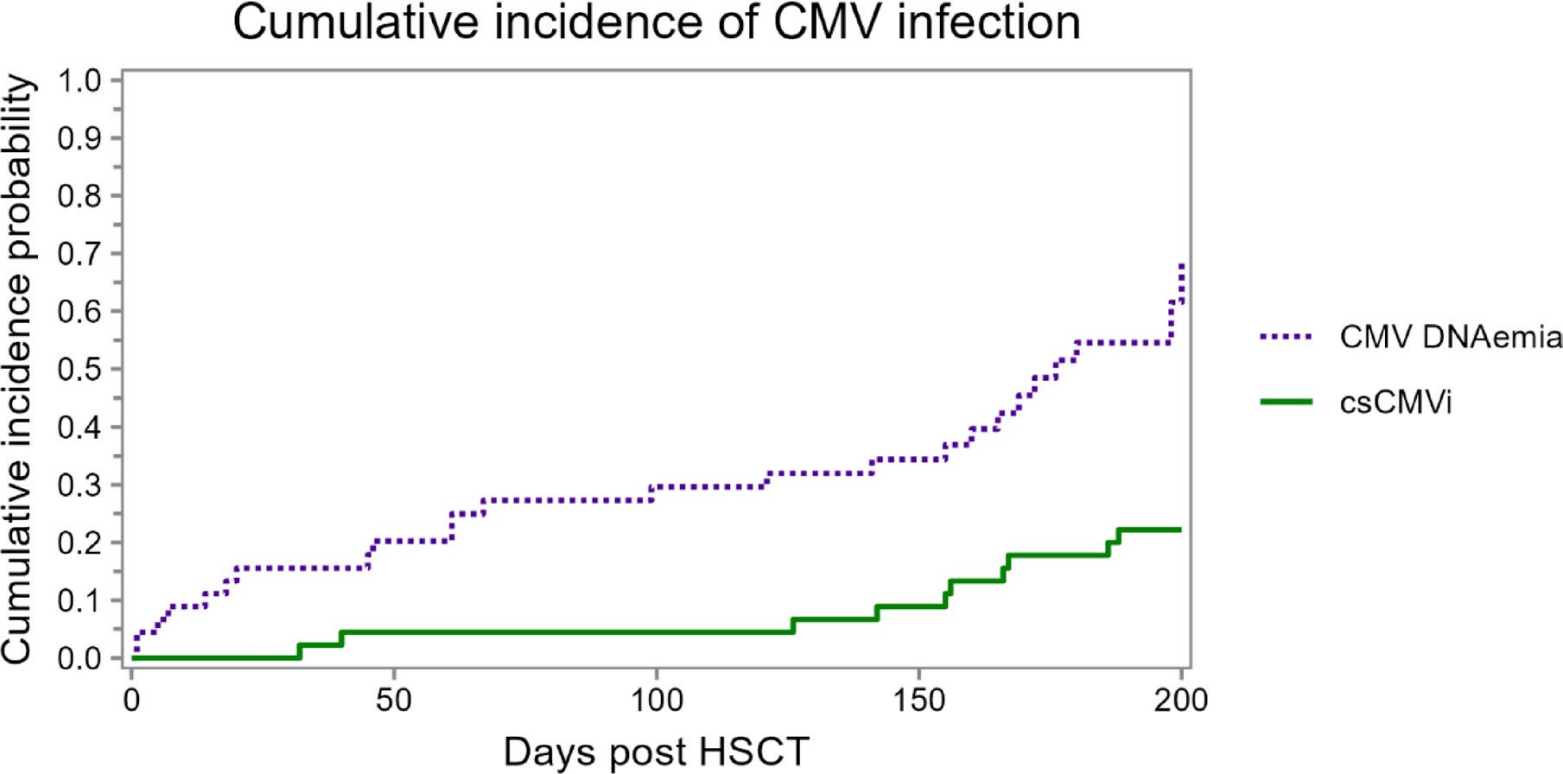
Cumulative incidence of CMV infection after allo-HSCT. Cumulative incidence of CMV DNAemia (blue) and csCMVi (green) up to day 200 post-HSCT. CMV, cytomegalovirus; csCMVi, clinically significant CMV infection; LTV, letermovir; HSCT, hematopoietic stem-cell transplantation.

The cumulative incidence of CMV DNAemia and clinically significant CMV infection (csCMVi) during the 200 days post-HSCT is shown in Fig. 2. While the overall probability of CMV DNAemia increased steadily over time, the cumulative incidence of csCMVi remained low during letermovir prophylaxis and began to rise predominantly after day 100, coinciding with discontinuation of prophylaxis. During the 12-month observation period, 39 of 45 patients (86.6%) experienced episodes of CMV DNAemia. Eighteen of these episodes (46.1%) occurred during letermovir prophylaxis, while 21 (53.8%) occurred after its discontinuation. Peak CMV DNAemia levels were significantly lower during letermovir prophylaxis compared to the post-prophylaxis period (median 89.5 IU/ml, IQR 64.3–130.0 vs. 150.0 IU/ml, IQR 89.5–445.5; *p* = 0.0403; Supplemental Fig. 1). For clinically significant CMV infections (csCMVi), median CMV DNA levels were 562.5 IU/mL (IQR 356.3–778.8) during letermovir prophylaxis and 355 IU/mL (IQR 215.5–825.0) after its discontinuation. Most early CMV DNAemia episodes under letermovir were low-level and transient, while clinically significant CMV infections (csCMVi, shown in orange) occurred predominantly after prophylaxis discontinuation and were associated with higher viral loads. The incidence of csCMVi was lower during letermovir prophylaxis (2/18, 11.1%) than in the post prophylaxis period (8/21, 38.1%). Among seven patients with recurrent CMV infections post-prophylaxis, six (85.7%) developed csCMVi. The median duration of antiviral treatment was similar between both groups: 65 days (IQR: 48.5–81-5) during prophylaxis and 45 days (IQR: 36–123.3) thereafter.

**Fig. 3:**
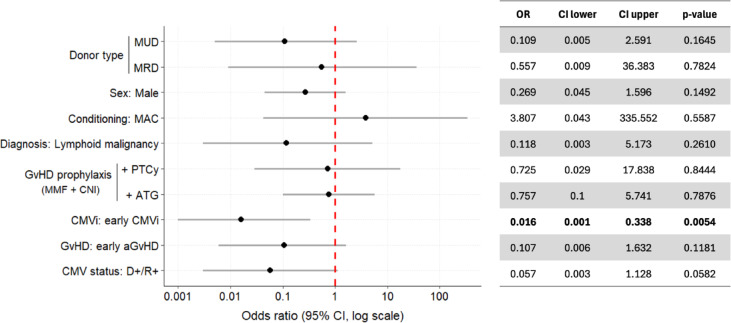
Univariate logistic regression analysis of clinical and transplant-related variables associated with late CMV infection. Forest Plot of Odds Ratios and 95% Confidence Intervals (95% CI). Early CMVi was defined as any CMV event within the first 100 days during letermovir prophylaxis. The analysis was conducted in the entire study cohort. ATG, anti-thymocyte globulin; CMVi, CMV infection; CNI, calcineurin inhibitor; D, donor; GvHD, graft-versus-host disease; MAC, myeloablative conditioning; MMF, mycophenolat-mofetil; MRD, matched related donor; MUD, matched unrelated donor; PTCy, post-transplant cyclophosphamide; R, recipient; RIC, reduced-intensity conditioning. Reference categories: Haploidentical (Donor type), female (sex), RIC (conditioning regimen), MRD (donor type), myeloid malignancy (diagnosis), no early CMVi (CMVi); no early aGvHD (GvHD); MMF + CNI (GvHD prophylaxis), D-/R + (CMV status).

To identify clinical and transplant-related factors associated with late CMV infection, exploratory univariate logistic regression analyses were performed (Fig. 3). Variables included early CMV infection during letermovir prophylaxis, donor-recipient CMV serostatus, donor type (related or unrelated vs. haploidentical), conditioning intensity (MAC vs. RIC), GvHD prophylaxis strategy (MMF + CNI + ATG or + PTCy vs. MMF + CNI only), occurrence of acute GvHD, patient sex, and underlying disease (myeloid vs. lymphoid malignancy). For categorical variables with more than two categories, dummy coding was applied, using haploidentical donor and MMF + CNI as the respective reference groups.

In this analysis, early CMV infection during letermovir prophylaxis was significantly associated with a reduced risk of late CMV infection (OR 0.016, 95% CI 0.001–0.338; *p* = 0.0054). No other variable showed a statistically significant association (Fig. 3). Donor-recipient CMV serostatus and GvHD prophylaxis showed numerical but non-significant trends consistent with previously reported risk factors.

**Fig. 4:**
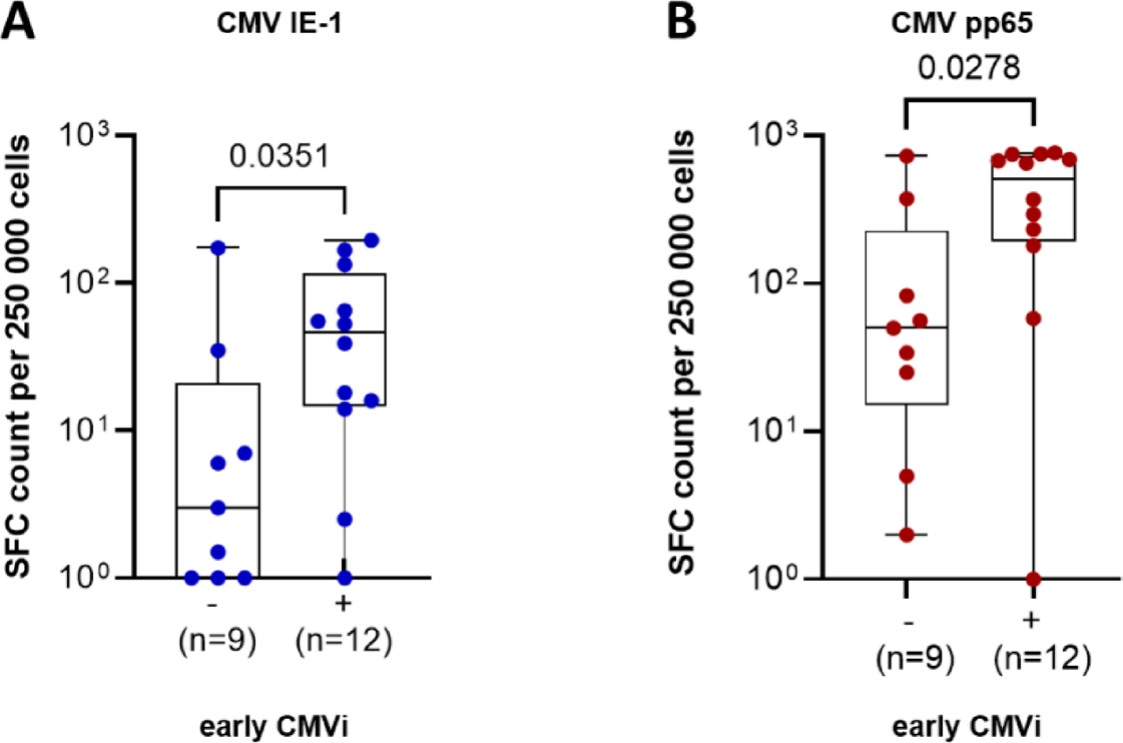
CMV-specific T-cell immunity at day 100 stratified by early CMVi during letermovir prophylaxis. Comparison of CMV-specific T-cell responses to the IE-1 antigen (**A**, blue) and the pp65 antigen (**B**, red) at Day 100 post-HSCT in patients with or without CMV infection during L´letermovir prophylaxis. Responses are expressed as spot-forming cells (SFC) per 250,000 PBMCs. Statistical comparisons were performed using the Mann–Whitney U test; *p*-values are shown above the plots. CMV, cytomegalovirus; HSCT, hematopoietic stem cell transplantation; PBMCs, peripheral blood mononuclear cells; SFC, spot-forming cells.

To investigate the immunological correlates of this protection, CMV-specific T-cell immunity was assessed using IFN-γ ELISpot assays at day 100 post-HSCT in 21 patients (Fig. 4).

IE-1-specific responses varied widely across the cohort, with a median of 16.0 SFC/250,000 PBMCs (IQR 2.25–46.0). In comparison, pp65-specific responses were consistently higher, with a median of 233.0 SFC (IQR 50.0–511.0). These data suggest that while pp65 responses are more robust overall, IE-1-specific responses may offer greater discriminatory value regarding individual risk.

When stratified by early CMV infection status (≤ day 100), patients with CMV DNAemia during letermovir prophylaxis had significantly stronger CMV-specific T-cell responses at day 100 post-transplant than those without early infection. For IE-1, the median response was 46.0 SFC (IQR 14.5–116.8) compared to 2.25 SFC (IQR 1.0–21.0; *p* = 0.0351). For pp65, the corresponding medians were 511.0 (IQR 193.3–732.8) vs. 50.0 (IQR 15.0–228.5; *p* = 0.0278) (Fig. 4A–B).

**Fig. 5:**
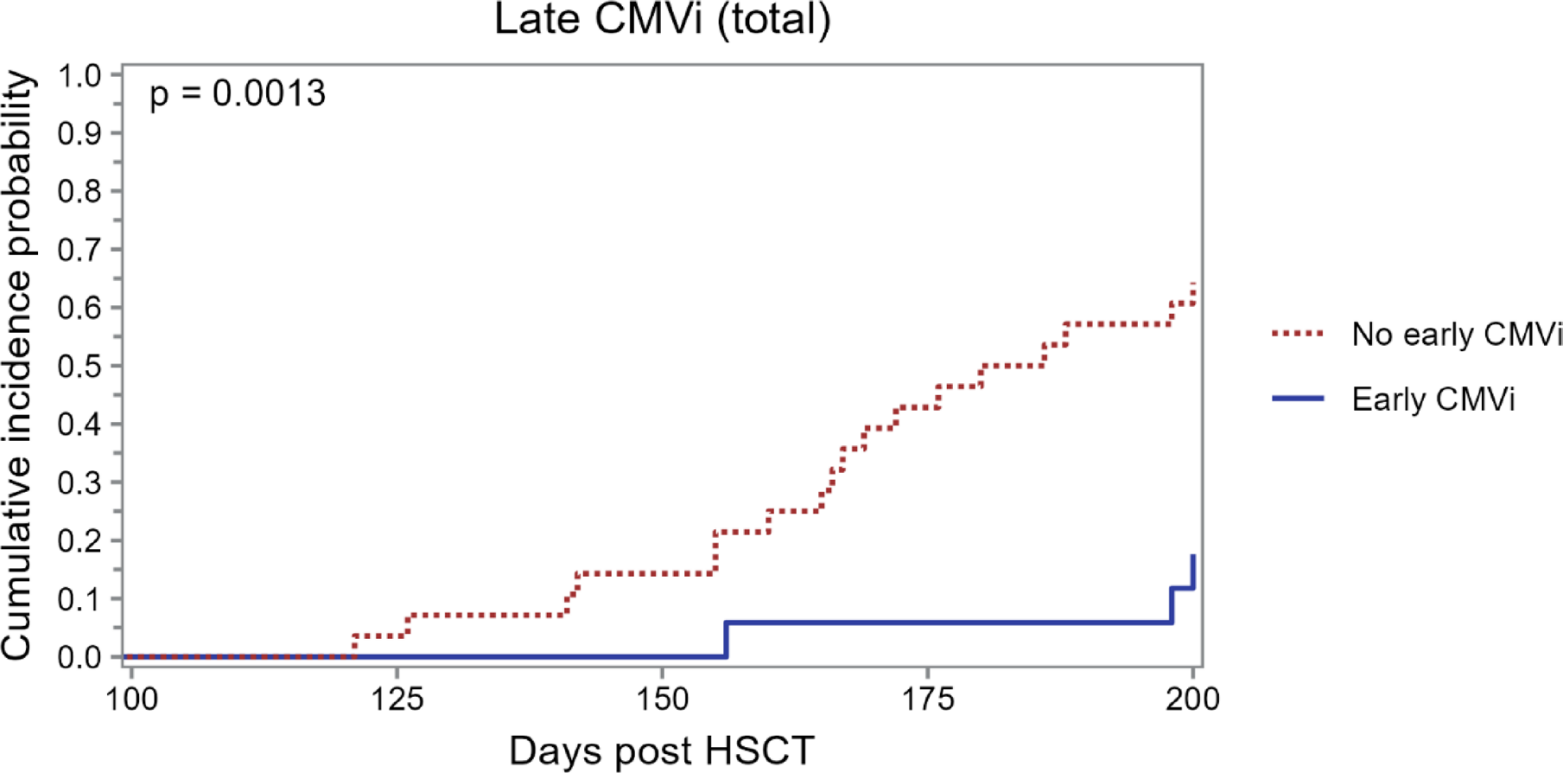
Cumulative incidence of late CMV infection stratified by early CMV status. Cumulative incidence of late CMV infection (day 100–200 post-HSCT) in the entire study cohort (n = 45), stratified by the presence or absence of CMV DNAemia during the first 100 days post-transplant (early CMVi). CMVi, cytomegalovirus infection; HSCT, hematopoietic stem cell transplantation.

To evaluate the clinical relevance of our immunological findings, we analyzed the cumulative incidence of late CMV infection (day 100 to 200 post-HSCT) in relation to early CMV infection. Notably, patients who experienced early CMV infection during letermovir prophylaxis showed a significantly lower rate of late CMV infection compared to those without early exposure (2/21 vs. 18/21; *p* = 0.0013, Fig. 5). When the analysis was restricted to clinically significant CMV infections (csCMVi, n = 10), similar trends were observed, although statistical significance was not reached due to the limited number of events (*p* = 0.0839; Supplemental Fig. 2).

To further explore the predictive value of these detected T cell immune responses, ROC analyses for both IE-1 and pp65 ELISpot results at day 100 were performed. While only IE-1 responses allowed the identification of a meaningful threshold, pp65 responses showed lower discriminatory power. For IE-1, a cut-off of 15 SFC per 250,000 PBMCs yielded an AUC of 0.768 (*p* = 0.0393) with a sensitivity of 88.9% (95% CI 56.5–99.4%) and specificity of 83.3%. The corresponding negative predictive value was 93.4% and the positive predictive value 79.2%, indicating good accuracy in predicting late CMV infection (Supplemental Fig. 3). In contrast, the ROC analysis for pp65-specific responses yielded an AUC of 0.536 (*p* = 0.778), indicating poor discriminatory performance. Using the cutoff of 67 SFC/250,000 PBMCs as proposed by Zamora et al., sensitivity was 60.0% (95% CI 31.3–83.2) and specificity 36.4%, resulting in a positive predictive value of 21% and a negative predictive value of 76%. Given these results, no clinically meaningful threshold for pp65 could be established in our cohort.

To further examine this threshold in a categorical framework, we evaluated the association of day 100 IE-1 ELISpot responses with subsequent events. Given the limited number of clinically significant infections, cumulative-incidence curves stratified by T-cell response were not generated to avoid overinterpretation. Instead, results are summarized in Table 2 using a contingency analysis based on the predefined threshold of > 15 IE-1 SFCs per 250,000 PBMCs. Late CMV infection occurred in 9 of 13 patients (69.2%) with low IE-1 responses but in only 1 of 12 (8.3%) with high responses (*p* = 0.0008; Fisher’s exact test), confirming the strong inverse association between detectable CMV-specific T-cell immunity and late CMV reactivation.

**Table 2:** Correlation of early CMV infection and day 100 IE-1 responses with late CMV infection. Contingency analysis evaluating the combined impact of early CMV infection and IE-1-specific T-cell responses at day 100 on the risk of late CMV infection. Section A displays the stratified contingency table; Section B summarizes post hoc subgroup comparisons. *P*-values were calculated using Fisher’s exact test. CMVi, cytomegalovirus infection; IE-1, immediate early 1 antigen; nc, not considered.

A. Combined stratification by early CMVi and IE-1 response			
Early CMVi	IE-1 > 15	Late CMVi (n)	No Late CMVi (n)
Yes	Yes	0	9
Yes	No	0	0
No	Yes	1	2
No	No	8	2
		Fisher’s exact test	***p*** **= 0.0008**

B. Post hoc comparisons between subgroups		
Comparison	Late CMVi rate	*p*-value
No early CMVi vs. early CMVi, all IE-1 levels	9/12 (75%) vs. 0/9 (0%)	**0.001**
Early CMVi with IE-1 > 15 vs. ≤ 15	0/9 vs. 0/0	0.999
No early CMVi with IE-1 > 15 vs. ≤ 15	1/2 (50%) vs. 8/10 (80%)	0.454

Significant values are in bold.

To validate these findings in a combined model, we performed a contingency analysis integrating early CMV infection status and IE-1-specific T-cell responses above the predefined threshold (Table 2A). None of the patients with an early CMV infection and an IE-1 response above the threshold developed late CMV infection. Post hoc comparisons (Table 2B) confirmed significantly higher rates of late CMV infection in patients without early CMV infection compared to those with early CMV events (9/12 vs. 0/9; *p* = 0.001). When stratifying within subgroups by IE-1-specific response level alone, no significant differences were observed.

**Fig. 6:**
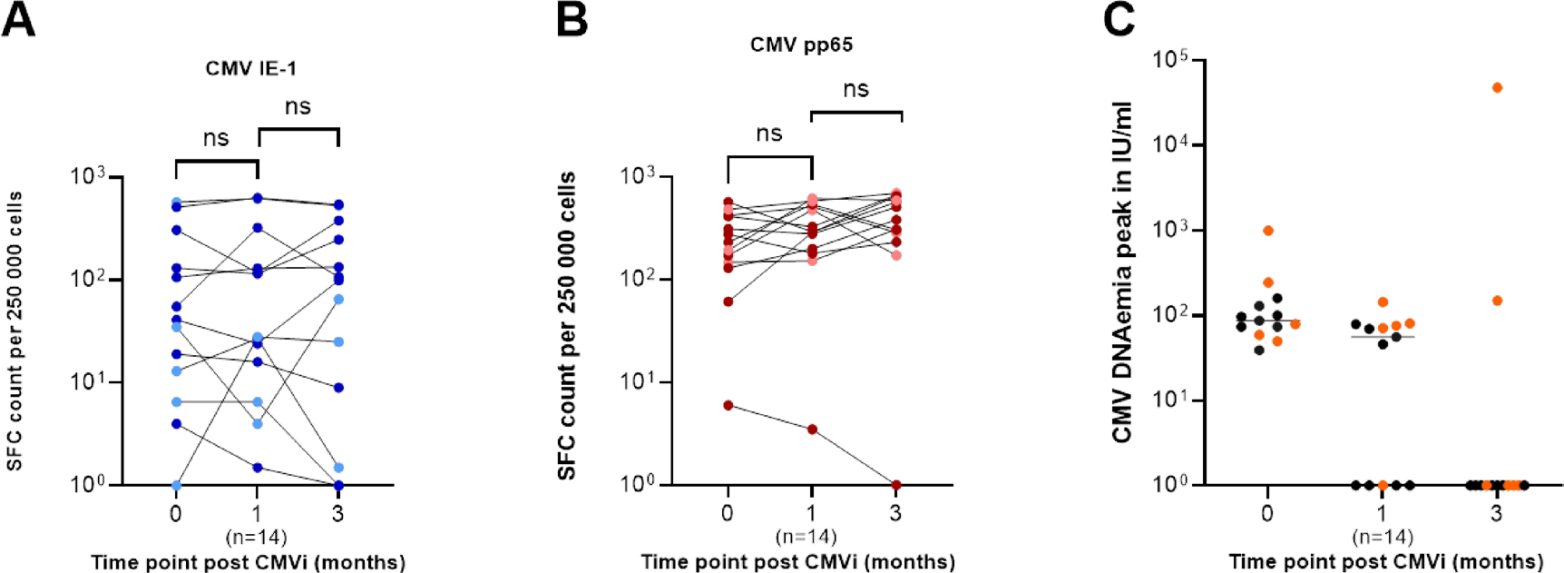
Longitudinal CMV-specific T-cell responses and viral load dynamics following CMV infection. (**A**–**B**) ELISpot of IE-1– (blue) and pp65–specific (red) T-cell responses at infection and after one and three months, shown as spot-forming cells (SFC) per 250,000 PBMCs. CsCMVi cases are highlighted in lighter colours. (**C**) CMV DNAemia peaks (black) and csCMVi episodes (orange) at corresponding time points; horizontal lines indicate medians. Mann–Whitney U test; ns = not significant. ELISpot, enzyme-linked immunospot; SFC, spot-forming cells; PBMCs, peripheral blood mononuclear cells; csCMVi, clinically significant CMV infection.

Finally, longitudinal follow-up of ELISpot responses after CMV infection, available for 13 study participants, showed that CMV-specific T-cell responses against both IE-1 and pp65 were already detectable at the time of infection and remained overall stable during the following three months (Fig. 6A,B). Although some individual variation was observed, including modest increases or occasional declines, there was no consistent or statistically significant change between the time of infection and the one- or three-month follow-up. Among the 13 longitudinally followed patients, 5 (38.5%) developed csCMVi. Median ELISpot responses and IQR for IE-1 were 41 (9.8–218.5) at the time of infection, 28 (11.3–228.8) one month later, and 99 (5.3–313.5) at three months. Corresponding pp65 responses were 233 (138.8–438.5), 293.0 (190.5–527.5), and 383 (258.8–622.0), respectively. Despite these variations, the overall kinetics indicated stable CMV-specific T-cell responses over time.

Corresponding viral load data for the same patients are shown in Fig. 6C. Median CMV DNAemia levels declined over time, from 87 IU/mL (IQR 66.5–145; range 39–995) at the time of infection to 56 IU/mL (IQR 0–77.5; range 0–144) after one month, and 0 IU/ml (IQR 0; range 0–48,000) after three months. At one month after CMV infection, 5 of 13 patients (38.5%) already showed complete viral suppression, and by three months, 11 of 13 (84.6%) were below the detection limit. Two patients exhibited persistent viremia (48,000 and 150 IU/mL, respectively), both corresponding to cases of clinically significant CMV infection (csCMVi). Detailed clinical and virological information for these 13 patients is provided in Supplemental Table 1.

In contrast, when comparing all available ELISpot results from day 100 and day 200, CMV-specific T-cell responses increased significantly over time. Median IE 1 responses rose from 16 to 133 SFC (*p* = 0.0025), and pp65 responses from 206 to 519 SFC (*p* = 0.0484) (Supplemental Fig. 4).

## Discussion

The management of CMV infection in immunocompromised patients, particularly in the setting of letermovir prophylaxis, remains a clinical challenge. While letermovir effectively prevents early-onset CMV reactivation, the risk of late CMV infection after discontinuation remains substantial. The role of CMV-specific T-cell immunity in this context has been well established^11,15,16^, and various studies in the pre-letermovir era have demonstrated the utility of immunoassays such as the ELISpot for risk stratification^16,18,19,23^.

In our exploratory logistic regression analysis, early CMV infection during letermovir prophylaxis was associated with a lower likelihood of subsequent late CMV infection. This observation may indicate that limited viral replication during prophylaxis is sufficient to prime CMV-specific immune responses without leading to clinically significant infection. Similar associations between early subclinical viral replication and improved CMV-specific immunity have been reported in other transplant cohorts^24,25^. However, due to the small number of late CMV infection events and the univariate nature of our analysis, these findings must be interpreted cautiously and cannot establish causality. Larger, prospective studies are required to confirm whether early CMV DNAemia indeed contributes to immune priming and subsequent protection against clinically significant infection.

In contrast to studies conducted before the introduction of letermovir, where early CMV reactivation was identified as a risk factor for subsequent late infection^1,26,27^, our findings likely reflect the modifying effect of letermovir prophylaxis. In our cohort, early CMV DNAemia was characterized by low viral loads and transient kinetics, without progression to clinically significant CMV infection (csCMVi). Similar patterns of low-level, self-limiting CMV DNAemia under letermovir have been described by Hakki et al. and Zavaglio et al.^28,29^. Such abortive replication under letermovir may provide limited antigen exposure sufficient for immune priming, while effectively preventing uncontrolled viral replication. This interpretation is consistent with the pharmacologic mechanism of letermovir, which inhibits the CMV terminase complex, leading to non-productive replication intermediates^3^, and with recent studies showing that transient CMV DNAemia under letermovir may contribute to immune priming and protection against late infection^18^.

We observed a significant increase in ELISpot responses between day 100 and day 200 across the cohort, indicating ongoing immune reconstitution. This finding is consistent with previous studies demonstrating that letermovir delays CMV-specific T-cell recovery after alloHSCT^2,13^. This longitudinal increase highlights the dynamic nature of post-transplant immune recovery, independent of prior CMVi status. Importantly, despite their lower numerical magnitude, IE-1-specific responses proved to be of greater clinical relevance for predicting late CMV infection.

However, the key prognostic information was derived from functional immunity assessed at day 100. Patients with early CMV infection during letermovir prophylaxis had significantly higher T-cell responses to both IE-1 and pp65. Importantly, stronger IE-1 responses at day 100 were associated with a lower risk of late CMV infection, while pp65 responses lacked such predictive value. These results suggest that even low-level CMV exposure during letermovir prophylaxis is sufficient to stimulate detectable and robust CMV-specific T-cell responses.

Our results further demonstrate that the strength of IE-1–specific T-cell immunity at day 100 is a key determinant of protection against subsequent CMV infection. Using a predefined threshold of > 15 IE-1 SFCs per 250,000 PBMCs, we observed that patients with higher responses rarely experienced late CMV infection, confirming the discriminative power of IE-1–directed immunity. This finding is consistent with recent multicenter studies identifying IE-1 responses as superior to pp65 for predicting CMV risk after letermovir prophylaxis^18,29^.

Moreover, the combined analysis integrating early CMV infection status and IE-1 responses provides additional evidence that transient antigen exposure during letermovir prophylaxis may support immune priming. None of the patients with both an early CMV DNAemia and a high IE-1 response experienced late CMV infection. This combined viro-immunologic profile may thus identify patients who have developed sufficient CMV-specific immune protection and may not require prolonged prophylaxis.

These results support and extend the findings by Zamora et al.^18^, who demonstrated in a multicenter study that low ELISpot responses to IE-1 but not pp65 were predictive of late clinically significant CMV infection after prophylaxis discontinuation. Notably, the authors proposed a threshold of 4 IE-1 SFCs per 250,000 PBMCs for risk stratification, underscoring the relevance of IE-1-directed immunity as a biomarker. In line with these findings, our ROC analysis confirmed a good discriminatory performance for IE-1 (AUC = 0.768, *p* = 0.0393), whereas pp65 responses showed only marginal predictive value (AUC = 0.536, *p* = 0.778) at the cutoff suggested by Zamora et al. (67 SFC/250,000 PBMCs). Thus, IE-1 appears to be the more reliable marker for protective immunity, consistent with both our data and earlier observations in the pre-letermovir setting^17,18,29^.

Importantly, our study also highlights that CMV infection itself, even at low levels during prophylaxis, may serve as a sufficient antigenic trigger for protective immunity. All patients who experienced CMV DNAemia during letermovir prophylaxis remained free of late CMV infection, independent of their day 100 SFC count.

Building on these observations, we next evaluated the clinical relevance of our immunological findings by analyzing the cumulative incidence of late CMV infection (day 100 to 200 post-HSCT) in relation to early CMV infection. Notably, patients who experienced early CMV infection during letermovir prophylaxis showed a significantly lower rate of late CMV infection compared to those without early exposure.

In contrast, one patient with an IE-1 SFC count above the threshold of 15, but no early CMV infection, still developed late clinically significant CMV infection. This underscores the importance of actual antigen exposure, even without symptoms, and suggests that the occurrence of CMVi itself may be a stronger marker of immune readiness than the ELISpot result alone. Similar conclusions were drawn by Zamora et al., who identified early CMV reactivation as a predictor of CMV-specific protective immune imprinting^18^.

Interestingly, in our cohort, six out of seven patients with recurrent CMV infections after the end of prophylaxis developed clinically significant CMV infection. This suggests that recurrent CMV DNAemia may also serve as a potential clinical marker for inadequate immune control. While this association has not yet been systematically evaluated in larger letermovir-treated populations, earlier studies have shown that multiple episodes of CMV reactivation are linked to delayed T-cell reconstitution and a higher risk of progression to csCMVi^9,30^. Recent multicenter data by Zamora et al. also support the notion that persistent or recurrent CMV detection in plasma correlates with insufficient immune priming and increased vulnerability to late CMV disease^18^. These findings underline the importance of recognizing recurrence of CMV DNAemia as an additional risk marker, which warrants prospective evaluation.

Importantly, all six patients with recurrent CMV infections in our cohort were also diagnosed with GvHD and received systemic corticosteroid therapy with prednisolone. These findings suggests that the observed loss of immune control may not solely reflect insufficient priming, but rather a secondary impairment of otherwise functional immunity. CMV-specific immunity is known to be dynamic and vulnerable to external influences such as GvHD, intensified immunosuppressive therapy, and disease relapse. As described by Nesher et al.^17^, even patients with robust T-cell responses early after HSCT can lose immune control under high-dose corticosteroid treatment. In such settings, ELISpot results may no longer reflect current immune status, and restarting letermovir prophylaxis could be clinically justified. These cases highlight the need to interpret immune monitoring in the context of concurrent CMV infection. In addition, donor-recipient CMV serostatus may influence CMV risk and immune reconstitution after transplantation. Although the incidence of late CMV infection did not differ markedly between serostatus groups in our cohort, the descriptive subgroup analysis highlights differences in conditioning intensity, donor type, and GvHD occurrence that may contribute to heterogeneous CMV outcomes.

While the idea of extending letermovir prophylaxis to prevent late CMVi remains under discussion^31^, our results suggest that such an approach may not be necessary in patients with prior non-clinically significant CMV infection, as these individuals appear to develop protective immunity through endogenous viral priming. Conversely, in patients without CMVi during prophylaxis, an IE-1 SFC count below 15 at day 100 could serve as a useful criterion to identify those at higher risk and guide a decision to prolong prophylaxis. Immune monitoring at day 100 may thus provide actionable information in select patients^32,33^.

Contrary to the assumption that CMV-specific T-cell responses require extended time to mature after infection, our longitudinal analysis revealed that CMV-specific T-cell immunity was already present at the time of CMVi and remained largely stable during the subsequent three months. This observation was corroborated by detailed ELISpot data from 13 evaluable patients, including five with clinically significant CMV infection, demonstrating sustained IE-1- and pp65-specific responses irrespective of CMV disease status. Together, these findings indicate that immune priming occurs early and that established CMV-specific T-cell immunity can be maintained even under conditions of intermittent antigenic stimulation. Consequently, a prolonged “immune consolidation phase” before discontinuing letermovir prophylaxis may not be necessary in patients who show evidence of early immune activation.

Our study has limitations. It was conducted at a single center with a limited number of patients, and the observational design restricts causal inference. The timing and availability of ELISpot samples varied, and our observation period was limited to 12 months. Importantly, due to the small number of clinically significant CMV infections observed, no definite conclusions can be drawn about the predictive value of early CMV infection or immune markers specifically for late csCMVi. However, in exploratory subanalyses restricted to csCMVi (Supplemental Fig. 2), the same trend was observed, with lower incidence among patients with early CMV DNAemia during letermovir prophylaxis. This suggests that our overall findings remain consistent even when only clinically significant CMV events are considered, although statistical confirmation in larger cohorts will be required. Another important limitation is the relatively small cohort size and the incomplete availability of ELISpot data. Immune monitoring was feasible in approximately half of the patients, and longitudinal analyses could be performed in only 13 individuals. While this restricts statistical power and limits generalizability, the observed associations were internally consistent and align with findings from larger multicenter cohorts. Thus, our data should be regarded as exploratory but hypothesis-generating, warranting confirmation in prospective studies with standardized immunomonitoring. In addition, detailed grading of acute GvHD was not systematically available and could therefore not be included in the analysis, which may limit the assessment of its potential influence on CMV risk.

In conclusion, our data support a tailored approach to letermovir prophylaxis. The occurrence of transient CMV DNAemia during letermovir appears to induce sufficient CMV-specific T-cell immunity to protect against late infection. Routine immune monitoring may not be necessary in these patients. In contrast, patients without early CMV exposure may benefit from ELISpot testing at day 100, and an IE-1 response below 15 SFC may justify extended prophylaxis depending on the individual clinical situation. These findings argue for a selective, risk-adapted approach to post-HSCT CMV management guided by immunological and virological markers.

## Supplementary Information

Below is the link to the electronic supplementary material.

Supplementary Material 1

## Data Availability

Data is provided within the manuscript and supplementary information files.
