# Supplementary material for: Early CMV DNAemia during letermovir prophylaxis predicts lower risk of late CMV infection and is associated with enhanced T-cell immunity after alloHSCT
Source: Sci Rep. 2025 Nov 20;15:41154. doi: 10.1038/s41598-025-27490-z (PMC12635171; doi:10.1038/s41598-025-27490-z)
Supplement: Supplementary file 1 — Supplementary Material 1 [file 41598_2025_27490_MOESM1_ESM.pdf]

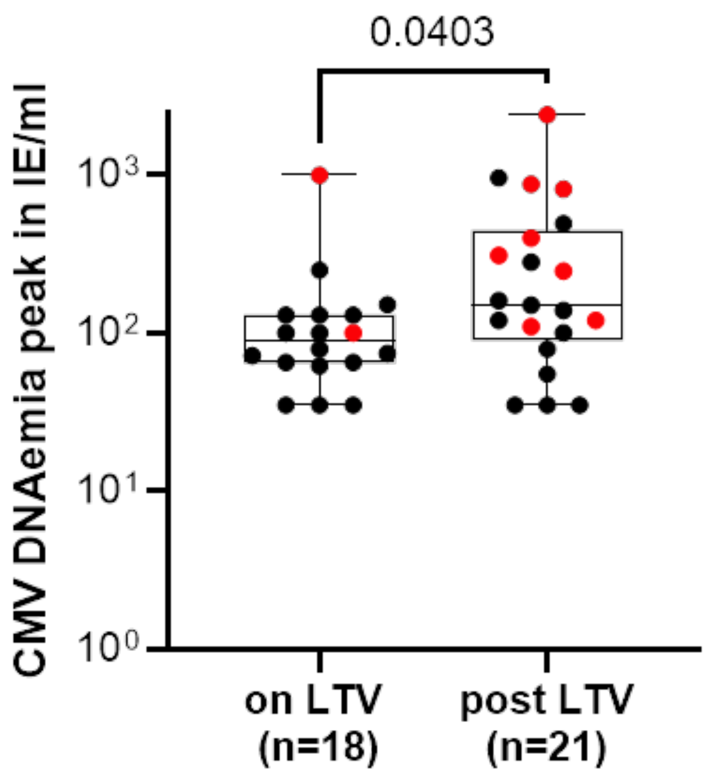

Supplemental Figure 1: Peak CMV-DNA plasma peak concentration during and after letermovir prophylaxis

Boxplots illustrating peak plasma CMV-DNA levels (IU/ml) in patients with any CMV event (n = 39), stratified by timing of infection in relation to letermovir prophylaxis: during prophylaxis (day 0–100) and after its discontinuation (day 100–200). Clinically significant CMV infection is indicated in red. CMV-DNA was quantified using real-time PCR.

Abbreviations: CMV, cytomegalovirus; IU, international units; PCR, polymerase chain reaction.

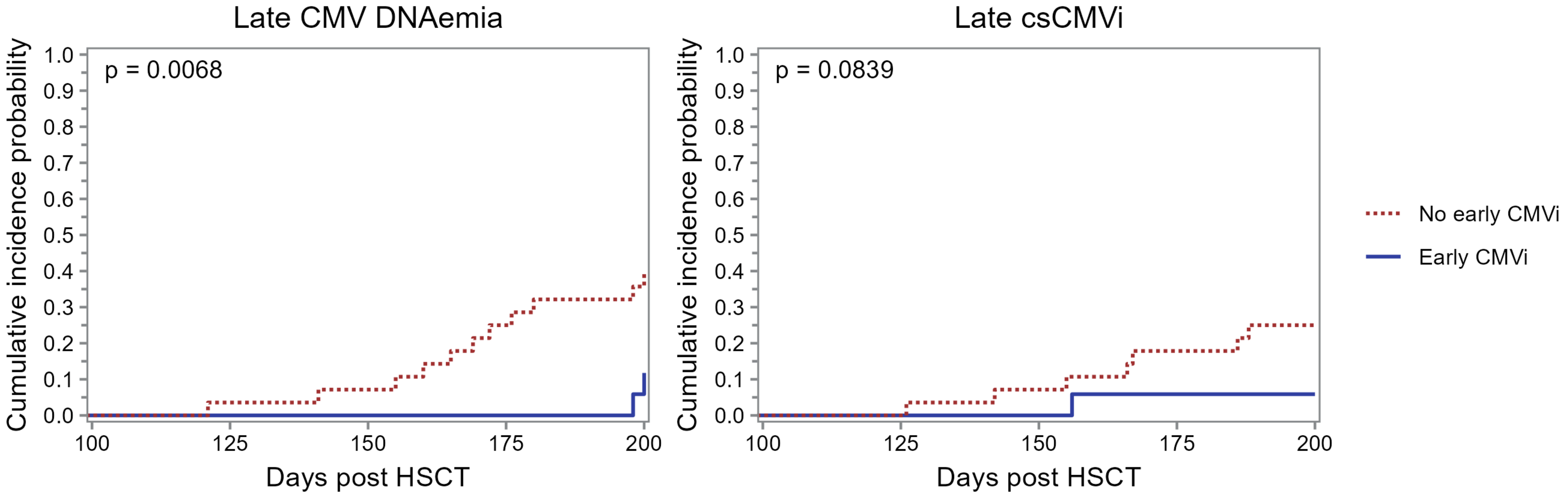

Supplemental Figure 2: Cumulative incidence of late CMV DNAemia and late clinically significant CMV infection stratified by early CMV infection
Cumulative incidence of late CMV DNAemia and late csCMVi (Day 100–200 post-HSCT) in the entire study cohort (n = 45), stratified by the presence or absence of CMV DNAemia during the first 100 days post-transplant (early CMVi).
Abbreviations: CMVi, cytomegalovirus infection; csCMVi, clinically signfifcant CMV infection; HSCT, hematopoietic stem cell transplantation.

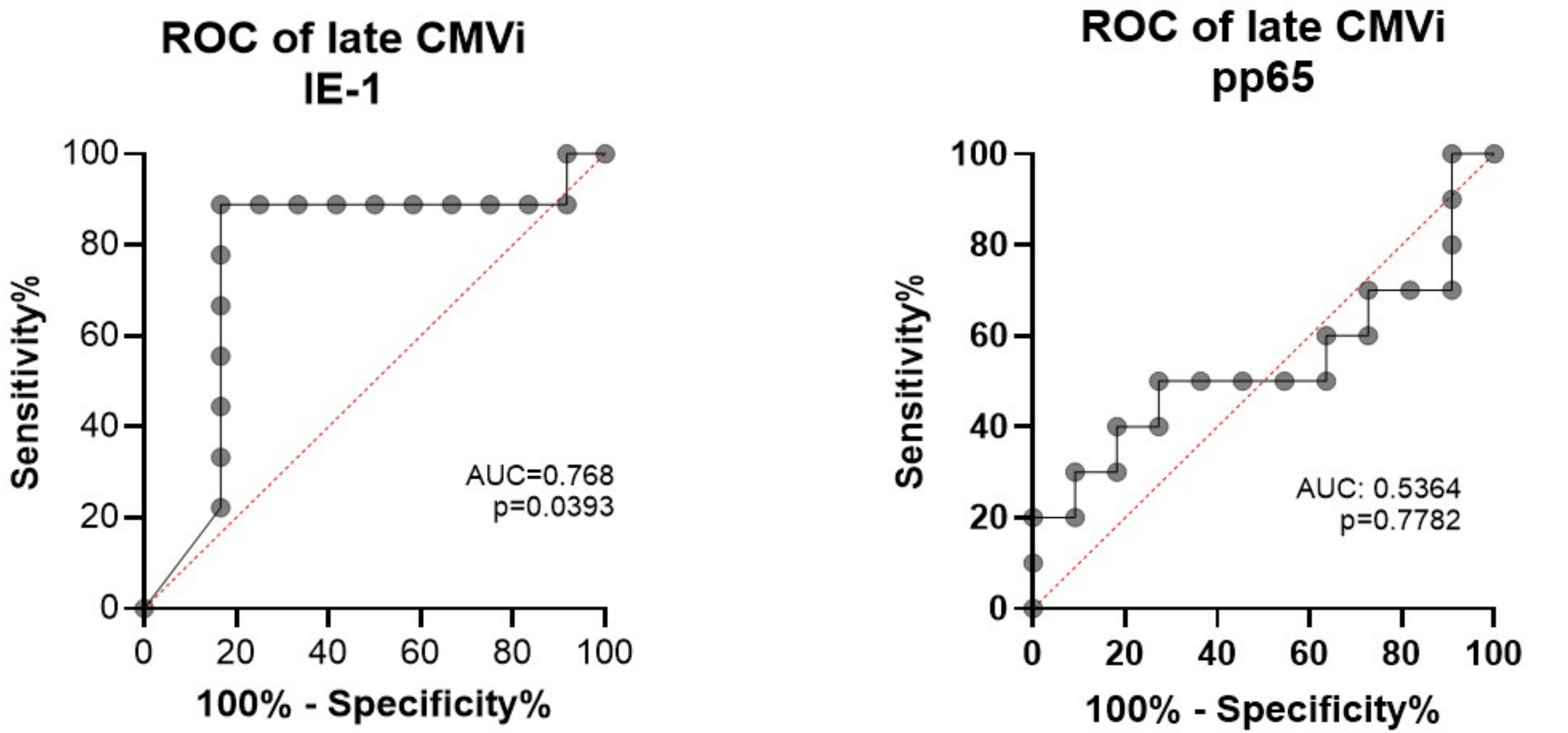

Supplemental Figure 3: ROC analysis of CMV-specific T-cell responses

ROC curves for IFN-γ ELISpot responses to IE-1 and pp65 antigens at Day 100 post-HSCT, evaluating their performance in predicting late CMV infection. The area under the curve (AUC) reflects the discriminatory ability of each antigen-specific response.

Abbreviations: ROC, receiver operating characteristic; AUC, area under the curve; IE-1, immediate early 1 antigen; CMV, cytomegalovirus; HSCT, hematopoietic stem cell transplantation.

Supplemental Table 1: Clinical and virological data of HSCT recipients with CMV infection and longitudinal ELISpot analysis

Shown are the timing of letermovir (LTV) prophylaxis, time to CMV infection, CMV-DNA levels at infection and during follow-up, and IE-1–specific T-cell responses measured by ELISpot at the time of CMVi.

Abbreviations: HSCT, hematopoietic stem cell transplantation; LTV, letermovir; CMVi, cytomegalovirus infection; csCMVi, clinically significant CMV infection; SFC, spot-forming cells; PBMC, peripheral blood mononuclear cells.

Patient ID	LTV start post-HSCT (day)	LTV discontinuation post-HSCT (days)	Time to CMV infection post-HSCT (days)	CMV-DNA at CMVi (IU/ml)	CMV-DNA at 1 month post CMVi (IU/ml)	CMV-DNA at 3 months post CMVi (IU/ml)	Clinically significant CMVi (csCMVi)	Duration of antiviral therapy (days)	IE-1–specific T-cell response at CMVi (SFC/2.5×10^5 PBMCs)
1	1	111	156	59	81	0	yes	106	13
2	1	104	126	50	71	0	yes	37	7
9	1	105	5	39	56	0	no	0	4
11	1	116	100	74	0	0	no	0	55
13	1	150	312	97	70	0	no	0	41
15	1	104	155	79	76	150	yes	129	572
16	1	119	200	100	0	0	no	0	306
18	1	121	198	150	46	0	no	0	511
19	1	123	198	74	79	0	no	0	132
22	1	112	167	148	0	0	yes	36	35
27	1	146	7	130	0	0	no	0	106
28	1	133	67	87	0	0	no	0	19
36	1	141	40	149	144	48 000	yes	98	1

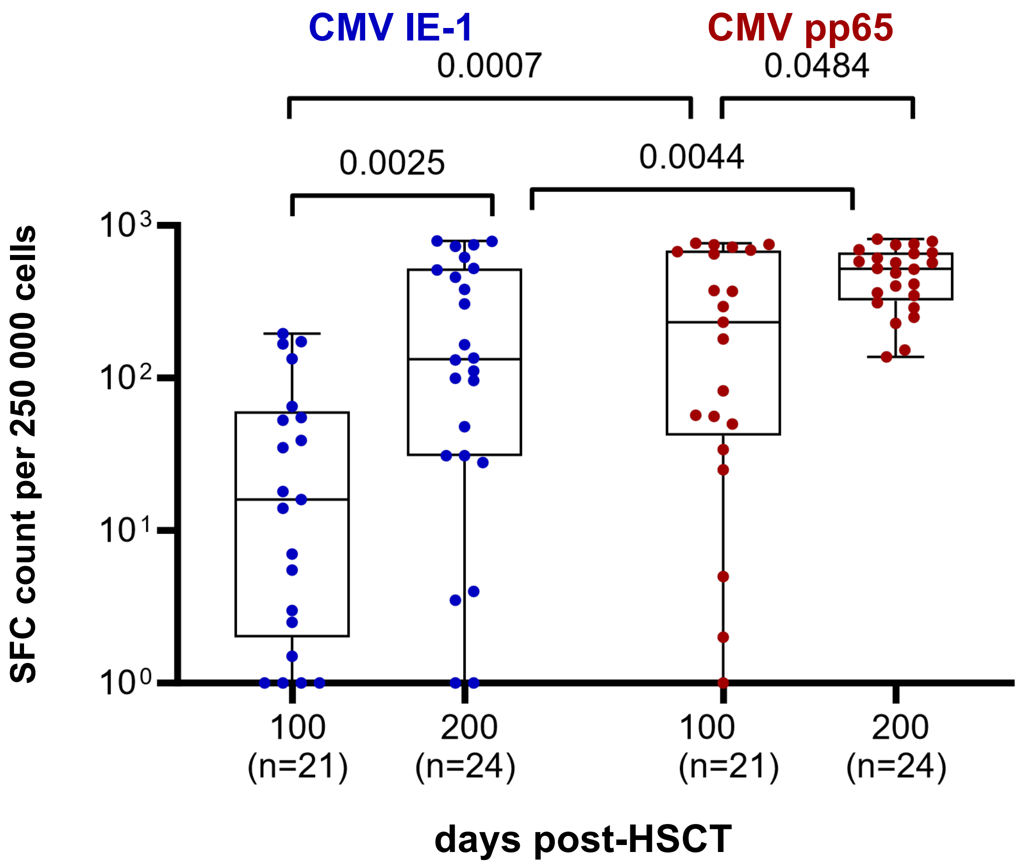

Supplemental Figure 4: CMV-specific T-cell responses at Day 100 and Day 200 post-HSCT

IFN-γ ELISpot responses to IE-1 and pp65 antigens at Day 100 and Day 200 after allogeneic HSCT. The figure includes all available samples at each time point; 19 patients contributed paired samples. T-cell responses are reported as spot-forming cells (SFC) per 250,000 PBMCs. Sample availability is summarized in Supplementary Table 1.

Abbreviations: CMV, cytomegalovirus; HSCT, hematopoietic stem cell transplantation; IE-1, immediate early 1 antigen; PBMCs, peripheral blood mononuclear cells; SFC, spot-forming cells; IFN-γ, interferon gamma.
